# Loss of Estrogen Receptors is Associated with Increased Tumor Aggression in Laryngeal Squamous Cell Carcinoma

**DOI:** 10.1038/s41598-020-60675-2

**Published:** 2020-03-06

**Authors:** Anjali Verma, Nofrat Schwartz, David J. Cohen, Vaidehi Patel, Benny Nageris, Gideon Bachar, Barbara. D. Boyan, Zvi Schwartz

**Affiliations:** 1 Department of Biomedical Engineering, Virginia Commonwealth University, Richmond, VA USA; 2 Department of Otolaryngology, Meir Hospital, Kfar Saba, Israel; 3 Sackler Faculty of Medicine, Tel Aviv University, Tel Aviv, Israel; 4 Department of Otolaryngology – Head and Neck Surgery and Neurosurgery, University of North Carolina School of Medicine, Chapel Hill, NC USA; 5 Department of Otolaryngology Head and Neck Surgery, Rabin Medical Center, Petah-Tikvah, Israel; 6 Department of Biomedical Engineering, Georgia Institute of Technology, Atlanta, GA USA; 7 Department of Periodontics, University of Texas Health Science Center at San Antonio, San Antonio, TX USA

**Keywords:** Steroid hormones, Steroid hormones, Steroid hormones, Prognostic markers, Prognostic markers

## Abstract

Laryngeal squamous cell carcinoma (LSCC) responds to 17β-estradiol via estrogen-receptor (ER, transcribed from ESR1) dependent mechanisms, but is not recognized as a hormonally responsive cancer. 17β-estradiol production by LSCC cell lines UM-SCC-11A and UM-SCC-12 was examined. Wild type (WT) and ESR1-silenced LSCC cultures and xenografts were examined for 17β-estradiol responsiveness *in vivo*. 14 LSCC and surrounding epithelial samples at various pathological stages were obtained from patients; ERα and ERβ expression were verified using data from the total cancer genome atlas. UM-SCC-11A and UM-SCC-12 both produce 17β-estradiol, but only UM-SCC-12, not UM-SCC-11A, xenograft tumors grow larger *in vivo* in response to systemic 17β-estradiol treatments. ERα66 and ERα36 expression inversely correlated with clinical cancer stage and tumor burden. LSCC ERα66 expression was higher compared to surrounding epithelia in indolent samples but lower in aggressive LSCC. ERβ expression was highly variable. High ESR1 expression correlated with improved survival in LSCC. Loss of ERα66 expression inversely correlated with prognosis in LSCC. ERα66 may be a histopathological marker of aggression in LSCC.

## Introduction

The larynx is an overlooked secondary sex hormone organ. Similar to other sex hormone organs, it undergoes trophic changes in response to hormonal changes during puberty, and morphological changes during adulthood. Steroid hormones have been reported to play a significant role in voice changes during maturation, as their effects mediate the lengthening and thickening of the male vocal folds^1^. In the female larynx, fluctuations in estrogen levels during the menstrual cycle are accompanied by variations in the pitch of the voice^2^. Atrophy and dystrophy have been found to be pronounced in the larynges of postmenopausal women as well as in the vocal fold tissue of ovariectomized rats^3^. These subjects are known to suffer from edema of the laryngeal lamina propria, inflammation in squamous and respiratory epithelia, pseudostratification, and cilia loss, but these symptoms were rectified with estrogen replacement therapy^4^.

Despite the obvious responsiveness of the larynx to estrogen, the presence and differential expression of estrogen receptors (ER) is a matter of debate, particularly in laryngeal cancer. ER, mainly ERα66, the traditional ER translated from the ESR1 gene, has been reported in laryngeal epithelia in both females^5^ and males^6^, and changes in ER are associated with inflammation and benign lesions^7^. ER expression has also been verified in laryngeal cancer^8,9^ and at higher levels than in adjacent normal mucosa^10^. Moreover, the non-traditional transmembrane ERs, G-protein-coupled receptor (GPR30) and ERα36, which is a splice variant of the traditional ERα66, have been found in human vocal folds^11^ and laryngeal cancer cells^12^, respectively. Another ER, ERβ, which is encoded by the ESR2 gene, has been inversely correlated with increased cancer aggression in laryngeal squamous cell carcinoma (LSCC)^13^. However, opposing studies have not identified ER^14^ or any other steroid hormone receptors^15,16^ in the larynx or laryngeal cancer cells^17^. The clinical implication of ER expression in laryngeal cancer is equally confounding. In a previous study, we found a correlation between the number of ERs and regional lymph node metastasis^12^. This differs from other studies that reported a reduced prognosis^18^ and lower rates of lymph node metastasis with increased ER expression^10^.

17β-estradiol (E_2_) is produced from testosterone by the enzyme aromatase during normal physiological function. At the cellular level, E_2_ elicits a wide array of nuclear and membrane signaling responses in different cells via different ERs. An assessment of estrogen’s full impact on laryngeal cancer must consider the vast number of nuclear, cytosolic, and membrane-associated ERs, the differential expression of ER in different cell types, and the convergence and crosstalk of the membrane and classical ER pathways in each cell. It is their integrative, at times synergistic or alternatively antagonistic, effects that confer the variability and complexity of E_2_ function in both normal and cancer cells^19^.

Estrogen exerts regionally specific effects on cell proliferation and mRNA expression of extracellular matrix (ECM)-associated genes in normal fibroblasts. Cervicothoracic fibroblasts from the vocal fold, trachea, and esophagus have been shown to respond differently to estrogen based on their specific estrogen profiles^20^. Studies examining the presence of ERα in vocal cord fibroblasts show that it is localized predominantly in the nucleus and cytoplasm^7^. Previous work by our group^12^ and others^20^ has shown that treatment with E_2_ suppresses ECM gene expression in laryngeal and vocal fold tissue, but through a membrane-associated ER rather than a nuclear ER. The complexity of E_2_ function and its dependency on local ER expression are further emphasized in our previous study with laryngeal cancer cell lines, in which we found E_2_ exhibited either a protective effect inhibiting DNA synthesis, or a deleterious effect augmenting proliferation and conferring anti-apoptotic potential to the cancer cells in a manner specific to the ER profile in each cell line^21^.

These findings stress the importance of further establishing the molecular and clinical characterization of laryngeal cancer in order to improve our understanding of the disease and its therapeutic options. The aim of our study was to further evaluate the different ER profiles that laryngeal cancer cells express and to correlate ER status with clinical prognoses. To better understand the role of ERα, we selected two closely related cell lines, both of which express the ERS1 gene. Whereas UM-SCC-12 cells produce ERα66 and ERα36, UM-SCC-11A cells produce only ERα36^21^. Furthermore, we implement our findings *in vitro* to evaluate how these cancers progress and behave with and without E_2_ in an animal model.

## Results

### UM-SCC-12 and UM-SCC-11A locally produce 17β-estradiol

**Figure 1:**
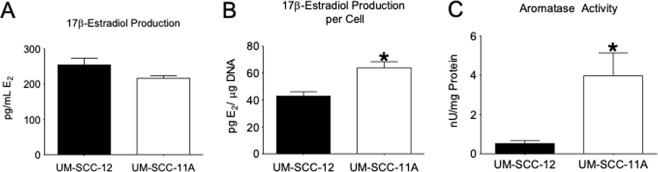
Panels A and B: Production of 17β-estradiol by ER+ UM-SCC-12 and ER− UM-SCC-11A per mL of cell supernatant (**A**) and per cell (**B**). Panel (C): Basal aromatase activity in laryngeal cancer cell lines. P-values less than 0.05 were considered significant. *Indicates significance against UM-SCC-12.

Both ERα66 positive (ER+) UM-SCC-12 cells and ERα66 negative (ER−) UM-SCC-11A cells produce similar concentrations (~250 pg/mL supernatant) of 17β-estradiol (Fig. 1A)^21^. However, ER− UM-SCC-11A produced ~1.5 times more 17β-estradiol per cell than their ER+ UM-SCC-12 counterparts (Fig. 1B). Similarly, UM-SCC-11A cells had higher basal aromatase activity than UM-SCC-12 cells (Fig. 1C), with approximately 4 nU of enzyme per mg of protein compared to UM-SCC-12’s 1 nU/mg protein. Figure 1B presents the data normalized to total number of cells (total DNA was used for normalization). The data in Fig. 1A indicate the total concentration of hormone produced by the cells.

### 17β-estradiol increased tumor aggression in estrogen receptor positive laryngeal cancer, but not estrogen receptor negative cancer *in vivo*

**Figure 2:**
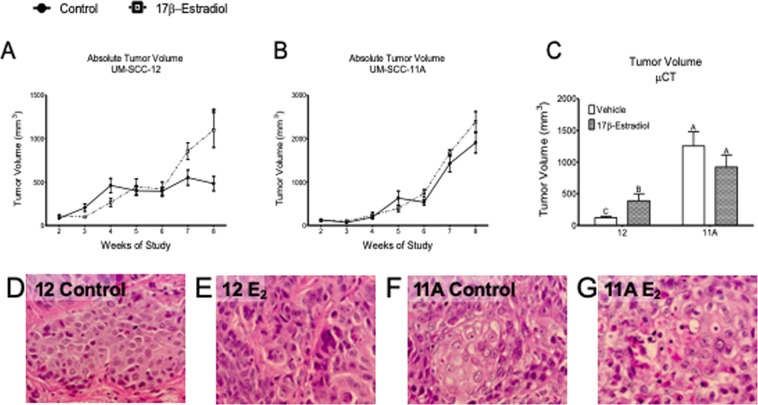
Panels (A,B): Effect of 17β-estradiol on tumor growth of ER+ and ER− xenografts *in vivo*. Tumor growth of UM-SCC-12 (ER+) (**A**) and UM-SCC-11A (**B**) over time with and without estradiol supplementation. *Indicates significance against week-matched control tumor volume. Panel (C): Final tumor volume was measured with μCT. Bars labeled with the same letter (‘A’) are not significantly different from each other (P > 0.05) but have values that are significantly different from bars labeled with different letters (‘B, C’). Panels (D–F): Representative histology of ER+ (**D**,**E**) and ER− tumors (**F**,**G**) with (**E**,**G**) and without 17β-estradiol (**D**,**F**) stained with hematoxylin and eosin and imaged at 40X.

**Figure 3:**
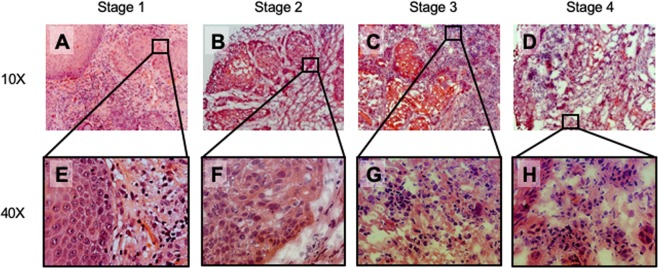
Representative histology of stage 1 (**A**,**E**), 2 (**B**,**F**), 3 (**C**,**G**), and 4 (**D**,**H**) LSCC. Images were taken at 10X (**A**–**D**) and 40X (**E**–**H**).

ERα66+ positive UM-SCC-12 xenografts grown in placebo-treated mice initially increased in volume (slope ≠ 0, Table S1), but did not increase in size after 4 weeks of growth (slope ≈ 0, Table S3) and ultimately reached a volume of 250–500 mm^3^ (Fig. 2A,C). In contrast, UM-SCC-12 xenografts grown in E_2_-treated mice increased in size over time (slope ≠ 0, Tables S1 and S3) and attained a final volume of 500–1000 mm^3^, 2-fold larger than tumors grown in control-treated mice (Fig. 2A,C). Most of this increase was observed after week 4. This effect was not observed in ERα66- UM-SCC-11A tumors, where both control and E_2_ treated tumors increased in size at a similar rate and to a similar final volume (Fig. 2B,C). Histology of UM-SCC-12 tumors revealed even cell shape, chromatin distribution, and uniform morphology in control tumors (Fig. 2D), similar to stage 1 LSCC (Fig. 3A,E). E_2_ treated tumors stained with haematoxylin and eosin (H&E) had similar eosin staining but darker haematoxylin staining, more irregular nuclei, and uneven chromatin distribution as compared to control treated tumors (Fig. 2E), similar to stage 2 LSCC (Fig. 3B,F).

### Estrogen receptor negative laryngeal cancer is more aggressive than estrogen receptor positive cancer

UM-SCC-11A subcutaneous xenografts implanted into mice grew ~250 times their original size to a final volume of 1000–2000 mm^3^ (Fig. 2B). UM-SCC-11A tumors attained a final volume 2–4 times greater than that observed in UM-SCC-12 xenografts (Fig. 2C). UM-SCC-11A tumors also grew at a continuous exponential rate throughout the study (R^2^ = 0.77–0.88, Table S2) with a faster doubling time than comparable UM-SCC-12 tumors (Table S2). Histology of UM-SCC-12 xenografts revealed moderately differentiated tumors with defined edges and uniform cell shapes (Fig. 2D,E). Conversely, histology of UM-SCC-11A xenografts revealed anaplastic tumors with high variability in nucleus and cell size within the tumor. These tumors also showed a significant amount of invasion into the surrounding tissue (Fig. 2F,G). The limited invasion and defined tumor edges observed in UM-SCC-12 cells are reminiscent of the defined tumor edges and even cell morphology observed in Stage 1 and 2 LSCC (Fig. 3A,B,E,F). The increase in invasion in UM-SCC-11A tumors as compared to UM-SCC-12 tumors is similar to the increase in tumor invasion observed in samples of stages 3 and 4 (Fig. 3C,D). The similar eosin staining, darker haematoxylin staining, irregular cell shape, and irregular nuclei structure observed in UM-SCC-11A tumors are similar to the cell morphology observed in stage 3 and 4 clinical LSCC samples (Fig. 3G,H). Estrogen receptor expression in clinical cases of laryngeal squamous cell carcinoma is highly variable and regionally localized.

Quantification of western blots showed that ERα66 (Figs. S1A and S2A), ERα36 (Figs. S1B and S2B), and ERβ (Figs. S1C and S2C) signal intensity varied exponentially from samples to sample. While all samples taken from stage 4 patients had similar total levels of ERα66 (Figs. S1A and S2A) regardless of locality, ERα36 and ERβ were highly variable and appeared to be regionally localized. Stage 4 samples collected at Meir Hospital, Israel (Table 1) had greater ERα36 staining (Figs. S1B and S2B) and ERβ staining (Figs. S1C and S2C) than samples collected at Massey Cancer Center, USA. All samples collected in Israel expressed high levels of ERβ, but none of the samples collected in the USA expressed ERβ (Figs. S1C and S2C). This regional variability was also apparent when considering the ratio of ER expression in tumor vs. precancerous tissue. All stage 3 and 4 samples collected in the USA had higher ratios of ERα36 in tumor tissue vs precancerous tissue (Table 2). However, stage 4 samples collected in Israel expressed less ERα36 in tumor tissue as compared to precancerous tissue (Tables 1 and 2). If neither tumor nor matched normal tissue expressed protein for ERα66, ERα36, or ERβ as measured by western blot, a value of ‘N/A’ was recorded for that sample (Table 2).

**Table 1:** Laryngeal squamous cell carcinoma samples and patient demographics.

ID number	Origin	Gender	Age	Location	Tobacco	Alcohol
1	Meir Hospital, Israel	M	78	glottic	1	0
2	Meir Hospital, Israel	M	76	glottic	1	1
3	Meir Hospital, Israel	M	68	glottic	1	0
4	Massey Cancer Center, USA	M	73	glottic	1	0
5	Massey Cancer Center, USA	M	57	glottic	1	1
6	Massey Cancer Center, USA	M	59	supraglottic	1	0
7	Massey Cancer Center, USA	F	67	supraglottic	0	0
8	Massey Cancer Center, USA	M	69	supraglottic	1	1
9	Massey Cancer Center, USA	M	64	glottic	1	1
10	Massey Cancer Center, USA	M	51	supraglottic	1	1
11	Massey Cancer Center, USA	M	64	supraglottic	1	0
12	Meir Hospital, Israel	M	58	supraglottic	1	0
13	Meir Hospital, Israel	M	70	glottic	1	0
14	Meir Hospital, Israel	M	65	glottic	1	0

**Table 2:** Estrogen receptor protein expression in clinical laryngeal squamous cell carcinoma samples.

ID number	Pathology	cT	cN	cM	Stage			
						ERα66	ERα36	ERβ
1	Invasive keratinizing SCC, NOS	1	0	0	1	↑	↑	↓
2	invasive keratinizing SCC, NOS	1	0	0	1	↑	N/A	↓
3	invasive keratinizing SCC, NOS	1	0	0	1	↑	N/A	↑
4	invasive keratinizing SCC moderate diff	2	x	x	2	↑	↓	N/A
5	invasive keratinizing SCC moderate diff	2	0	x	2	↓	↓	N/A
6	invasive keratinizing SCC moderate diff	3	0	x	3	↑	↑	N/A
7	invasive keratinizing SCC moderate diff	3	0	x	3	↑	↑	N/A
8	invasive keratinizing SCC moderate diff	3	0	x	3	↑	N/A	N/A
9	invasive keratinizing SCC moderate diff	4	x	x	4	↑	↑	N/A
10	invasive keratinizing SCC moderate to poorly diff	4	2b	x	4	↓	↑	N/A
11	invasive keratinizing SCC moderate diff	4	2c	x	4	↓	↑	N/A
12	invasive keratinizing SCC moderate diff	4	1	0	4	↓	↓	↓
13	invasive keratinizing SCC moderate to poorly diff	4	2	0	4	↓	↓	↓
14	invasive keratinizing SCC, NOS	4	0	0	4	↓	↓	↓

↑ indicates a ratio >1, ↓ indicates a ratio < 1.

### Decreased absolute and normalized ERα66 expression is associated with advanced cancer stage in clinical samples of laryngeal squamous cell carcinoma

**Figure 4:**
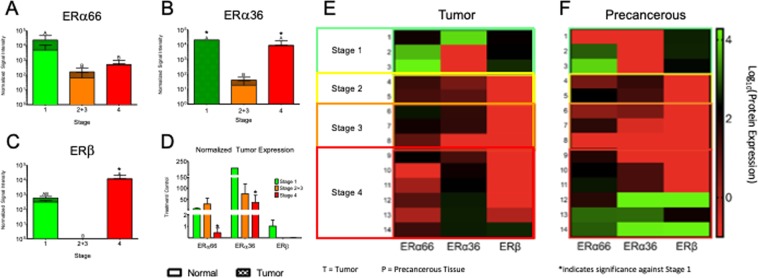
Panels (A–C): Quantification of western blots of ERα66 (**A**), ERα36 (**B**), and ERβ (**C**). Samples were grouped by cancer stage (stage 1, stages 2 and 3, stage 4) and analyzed by one-way ANOVA with Tukey’s correction. Bars not sharing a letter are significantly different from each other with P < 0.05. Bars are shown as total protein expression, with solid bars representing protein expression in normal epithelia and patterned bars representing protein expression in tumor tissue. Y-axes are given as log scales. Panel (D): Protein expression in LSCC samples was normalized to expression in surrounding epithelia, grouped by stage, and graphed. *Indicates significance against stage 1 within protein groups. Panels (E,F): Heat maps showing tumor (**E**) and surrounding epithelia (**F**) expression of ERα66, ERα36, and ERβ for each sample. Individual protein expression was measured by western blot and graphed as log_10_ of normalized signal intensity. Samples that did not express any protein were taken as ‘0’ expression and are represented by red panels on the heat map.

A general trend towards lower ERα66 expression was observed in more aggressive tumors (Stage 2–4) as compared to indolent tumors (Stage 1) (Fig. 4A,E). The total amount of ERα66 in these samples was greatest in stage 1 cancer (Fig. 4A), and the proportion of ERα66 expressed by the tumor tissue as compared to matched precancerous tissue was also greater in stage 1 samples as compared to stage 4 samples (Fig. 4D–F, Table 2). Samples from patients with stage 2–3 cancer had less ERα66 overall as compared to stage 1 samples (Fig. 4A) but, like stage 1 samples, tumor tissues expressed more ERα66 than matched precancerous tissue (Fig. 4D, Table 2). This trend did not hold true for Stage 4 cancer samples, where the ratio of ERα66 expression to precancerous tissue was flipped and precancerous tissue expressed more ERα66 than matched tumor tissue samples (Figs. 4A,D–F; Table 2).

Patterns of relative ER expression among cancer stages described for ERα66 above also held true for ERα36 and ERβ (Fig. 4B,C). Tumor tissue taken from patients with stage 4 cancer generally expressed less ERα36 and ERβ than matched precancerous samples (Fig. 4B–F, Table 2). The ratio of ER expression in tumor vs. precancerous tissue was significantly lower in stage 4 samples as compared to stage 1 samples for all ERs: ERα66, ERα36, and ERβ (Fig. 4D, Table 2).

Unlike ERα66, absolute expression of ERα36 and ERβ was similar in stage 1 and stage 4 cancers (Fig. 4B,C). The most aggressive cancers (stage 4) generally had low ERα66 expression and high ERα36 expression (Fig. S1A,B). Absolute ERα36 expression in stage 2 and 3 cancer samples was lower than stage 1, but ERα36 expression generally increased with stage in tumor samples taken from stage 2–4 patients (Fig. S1B). Normal tissue surrounding stage 4 samples expressed higher ERα36 than matched normal tissue from lower stage cancers. Neither tumor nor precancerous samples taken from stage 2 and 3 samples patients expressed ERβ. However, it is important to note that all stage 2 and 3 samples were collected in the USA, while stage 4 samples were collected in both the USA and Israel.

### Higher expression of ESR1 but not ESR2 is associated with increased survival in patients with primary laryngeal squamous cell carcinoma

**Figure 5:**
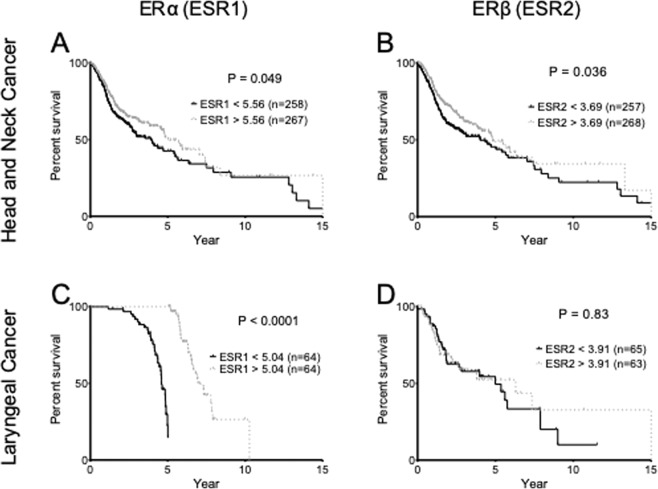
Kaplan-Meier survival curves for patients with any primary head and neck squamous cell carcinoma (**A**,**B**) or primary LSCC (**C**,**D**) stratified by higher or lower than median ESR1 (**A**,**C**) or ESR2 (**B**,**D**) expression. Survival curves were analyzed with Log-rank Mantel-Cox χ^2^ tests. P-values less than 0.05 were considered significant.

Analysis of RNAseq data taken from the TCGA study of 525 patients with primary head and neck cancer showed that patients with higher than median tumor ESR1 expression (ESR1> 5.56) had significantly better survival rates than patients with lower tumor ESR1 expression (Fig. 5A). This trend was also observed when patients were stratified by tumor ESR2 expression, with patients with higher than median ESR2 expression (ESR2 > 3.69) (Fig. 5B) having significantly higher survival rates than patients with lower ESR2 expression after 15 years.

RNAseq data taken from a TCGA study of 128 patients with primary LSCC showed that patients with higher than median tumor ESR1 expression (ESR1 > 5.56) had significantly higher survival rates as compared to patients with lower tumor ESR1 expression (Fig. 5C) with p-value < 0.0001 and a hazard ratio of 0.02845. However, no correlation between ESR2 expression and survival was observed when patients were stratified into groups of higher and lower than median ESR2 expression (Fig. 5D) (p-value = 0.83).

### Silencing ESR1 in estrogen receptor positive laryngeal squamous cell carcinoma eliminates the 17β-estradiol response and increases aggression

Silencing the ESR1 gene, which encodes all known ERα isoforms, in UM-SCC-12 cells reduced ERα66 expression by 76% as compared to a 33.5% knockdown in scramble control cells (Fig. S3A,B). Similarly ESR1 silencing knocked down ERα36 expression by 75.2% as compared to 44.4% knockdown in scramble control cells (Fig. S3A,C).

**Figure 6:**
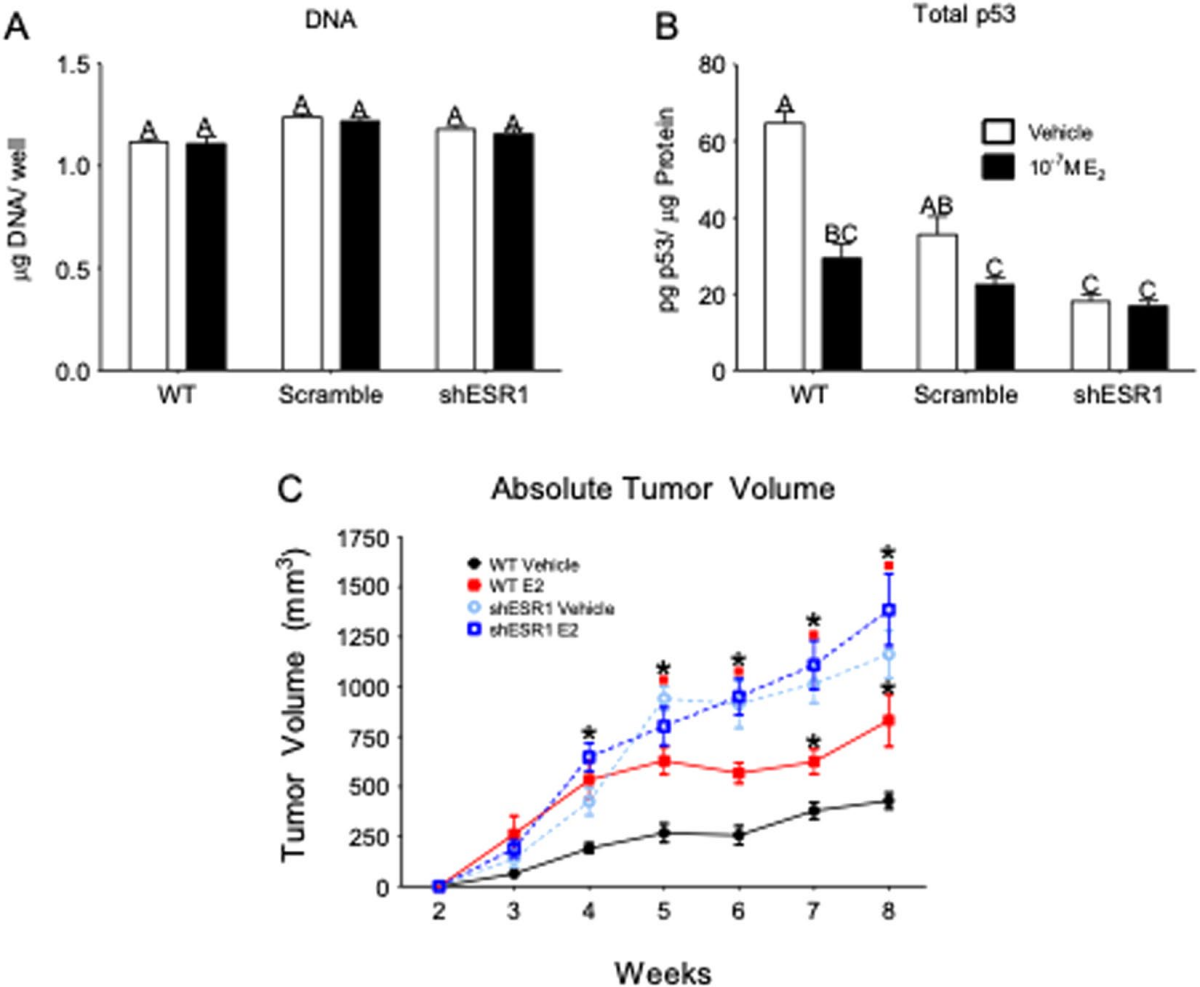
Effect of silencing ESR1 in ERα66+ UM-SCC-12 cells *in vitro* on cell number (**A**) and the response to estrogen as measured by total p53 (**B**). Bars that do not share a letter are considered significant with p-values less than 0.05. *In vivo*, WT and ESR1-silenced UM-SCC-12 cell-line xenografts were implanted in a subcutaneous xenograft mouse model and tumor burden was measured over time (**C**). Black * Indicates significance against week-matched WT-vehicle tumor volume. Red solid squares (no error bars)  indicate significance against week-matched WT-E_2_ tumor volumes. P-values less than 0.05 were considered significant.

Silencing ESR1 in UM-SCC-12 cells did not change cell number *in vitro* as measured by total double-stranded DNA content (Fig. 6A). However, silencing ESR1 altered total p53 content in these cells (Fig. 6B). p53 protein production is associated with apoptosis. Total p53 content was significantly lower for shESR1-UM-SCC-12 cells as compared to WT and scramble control UM-SCC-12 cultures. Furthermore, silencing ESR1 in UM-SCC-12 cells eliminated their response to E_2_. Both vehicle and E_2_-treated shESR1-UM-SCC-12 cultures had approximately 1/3 the p53 content of wild type (WT) vehicle-treated UM-SCC-12, whereas both vehicle and E_2_-treated shESR1-UM-SCC-12 cultures had levels of p53 similar to those observed in WT UM-SCC-12 cells treated with E_2_ (Fig. 6B).

UM-SCC-12 subcutaneous xenografts silenced for the ESR1 gene grew approximately 4 times larger than WT UM-SCC-12 xenograft tumors and twice as large as WT UM-SCC-12 xenografts treated with E_2_. shESR1-UM-SCC-12 xenografts grew to similar sizes regardless of E_2_ supplementation. Both silenced and WT xenografts grew in an approximately linear fashion, suggesting a faster doubling time *in vivo* for shESR1-UM-SCC-12 cells as compared to WT UM-SCC-12.

## Discussion

Accumulated evidence has substantiated that laryngeal cancer, a common head and neck cancer in the United States, is a hormone responsive cancer, comparable to other more renowned secondary sex hormone cancers. Despite the reports of E_2_ detrimental effects in laryngeal cancer^22,23^ and that anti-estrogen treatment has a beneficial effect^24^, originating almost three decades ago^25^, there has been little advance in translating this recognition of the importance of E_2_ to practical clinical implications. This might be explained by the confounding and heterogeneous ER profile detected in these cancer cells. The cumulative effects of this heterogeneity translate to disparate responses to E_2_ and must be clarified before implementation to clinical practice.

Local production of E_2_ has been described in many steroid hormone responsive cancers, including breast^26^, endometrium^27^, cervical^28^, and testicular cancer^29^. Here, we observed that both UM-SCC-12 and UM-SCC-11A cultures maintained a concentration of ~250 pg/mL of E_2_ in their surrounding media regardless of cell number. This concentration is roughly 20 times previously reported levels of estradiol in serum from healthy adult males^30^, but is not dissimilar from E_2_ levels reported in plasma from pre-menopausal breast cancer patients^31^. Similar levels of E_2_ production have also been observed in breast cancer-associated fibroblasts^32^. The tumorigenic properties of estrogen have been well-described in breast cancer^33,34^, and our previous work has shown that E_2_ is also tumorigenic in ERα66+, but not in ERα66-, laryngeal cancer^12,21^. The increase in aromatase activity and corresponding increase in E_2_ production per cell in ER− LSCC vs. ER+ LSCC were surprising; however similar disparities in E_2_ production have been observed in ER+ and ER− breast cancer^35^. Increased serum E_2_ is associated with compensatory mechanisms that arise in conjunction with defective estrogen signaling in normal breast tissue^36,37^, and elevated serum E_2_ has also been reported in estrogen insensitive triple-negative breast cancer (TNBC) patients as compared to those with ER+ tumors^35^. It is possible that the increase in aromatase activity and subsequent increase in E_2_ production per cell was due to a saturation in E_2_ concentration reminiscent of a classic negative signaling feedback loop^38^. It is also possible that the elevated E_2_ production and aromatase activity observed in ERα66- LSCC is a result of similar compensatory signaling associated with these cells’ insensitivity to estradiol^21^.

Consistent with previous reports^5–10^, evaluation of the laryngeal epithelia adjacent to laryngeal cancer reveals expression of both classical nuclear ERs and membrane ERs, specifically the ERα isoform ERα36. ERα36 is a well-established ERα splice variant that can mediate the effects of E_2_ independently of nuclear ERα. This effect has been demonstrated in ERα66 null cells and in studies using antibodies against the nuclear and membrane isoforms of ERα^39^. The receptor initiates divergent pathways from the plasma membrane that re-converge downstream to affect cancer cell survivability^40^. In our study a striking difference was evident between the epithelia adjacent to advanced aggressive tumors and to more indolent tumors. While ERβ expression was highly variable, both ERα variants, the classical nuclear ERα66 and ERα36, were expressed in high levels in the epithelia adjacent to tumors displaying aggressive behavior, while absent in the indolent samples. In this, the ERα expression patterns in the epithelia adjacent to aggressively malignant samples (stage 4) is reminiscent of the ERα66 and ERα36 expression profiles generally seen in more indolent tumor samples.

It is well known that laryngeal squamous cell carcinoma arises from precancerous lesions, mainly dysplastic lesions. However, the exact molecular mechanisms of malignant transformation of laryngeal mucosa are not clear. Moreover, the ability to identify patients who are most likely to progress into invasive laryngeal cancer or possess warning aggressive markers early in the course of their disease is paramount to early diagnosis and treatment to confer better prognoses. The presence of markers that correlate with pre-malignancy or early-stage cancer could have cardinal bearing on clinical decisions to observe or aggressively treat lesions.

Various classification systems have been crafted in an attempt to describe the histologic features of these laryngeal epithelial precursor lesions. Unfortunately, a universally accepted histopathological classification system and consensus on diagnostic criteria for LSCC are lacking. Different diagnostic techniques, specifically imaging modalities of laryngeal epithelial lesions, have been developed, but they do not offer a single system to make a differential diagnosis^41^. Consequently, new markers are required to reliably identify those high-risk precancerous lesions. Effort has been directed to identify molecular biomarkers that will enable the stratification of risk for malignant transformation and aggressive tumor behavior in patients with precancerous lesions. Some proposed biomarkers include the presence of chromosome instability markers^42^, cell cycle proteins^43^, β-catenin^44^, and microRNA-21^45^.

To our knowledge, ER characterization and profiling has not been studied in the context of precancerous lesions. Our findings imply that laryngeal epithelia expressing high levels of ERα66 and ERα36 are at risk for aggressive malignant transformation. Thus, patients expressing this phenotype should be considered for more aggressive treatment and followed closely. Our sample number is too small to draw conclusions; however, in this evolving field, ER status should be considered as another biomarker that can shed light on the malignant potential of the tumor and direct treatment.

Evaluation of advanced and aggressive tumors, with clinical stage 4, revealed recovered high rates of ERα36, while remarkably losing or reducing their expression of ERα66. Indolent tumors, clinical stage 1, unexpectedly were found to upregulate the expression of both ERα66 and ERα36 compared to the adjacent epithelia. The majority of stage 2 and 3 tumors had reduced ERα66 expression as compared to more indolent samples, but retained their overexpression of ERα66 as compared to adjacent tissue. Conversely, there was a general trend of increasing ERα36 expression in more aggressive samples, suggesting that it is only in the final most aggressive stage 4 that tumors begin to lose ERα66 and regain ERα36 expression.

In an attempt to interpret the differential ER expression between more indolent and aggressive tumors we turn to the prototype by which intricate ER cellular functions have been extensively studied, breast cancer. Nuclear ERα66 has been used as both a diagnostic and prognostic marker in breast cancer for many years. It’s presence can dictate clinical treatment, making the use of anti-estrogen therapies possible for patients with ERα66 overexpressing tumors. Typically, most of these tumors eventually become resistant to these therapies through acquired resistance or de-novo^46^. One prevailing theory for the resistance to anti-estrogen therapy is the loss of the expression of ERα66, either as the presenting phenotype in ERα66 negative tumors or in ERα66 positive tumors during treatment, imparting loss of constraints and deleterious effects on tumor behavior. ERα66 negative breast cancer, specifically the subclass of triple negative cancers, are renowned for their aggressive nature and grave malignant potential^47–49^. Thus, in the case of these most hormone responsive cancer, it is the loss of that responsiveness that causes the most aggressive behavior.

Interestingly, ERα66 positive breast cancer acquired loss of the expression of ER precipitates the same aggressive phenotype. That same loss of ERα66 expression has been described to incur trans-differentiation from epithelial to mesenchymal phenotype, which is responsible for increased aggressiveness and metastatic propensity^50^. More than a third of patients with recurrence and metastases from primary ERα66 positive breast cancer were found to have lost their expression of ERα66 upon recurrence^51,52^, which incurred a worse prognosis^53^. Coupled with the fact that almost half of patients with aggressive breast cancer characterized with high rates of recurrence and distant metastasis are classified as bearing primary triple negative cancers^54^, it seems evident that the lack of ERα66 expression confers poor prognosis.

That loss of ER confers a poorer prognosis was further evidenced in our animal model. While the subcutaneous xenograft model we used does not traditionally metastasize^55–57^, the increased aggression associated with low ER and E_2_ sensitivity in breast cancer was observed, with ER− LSCC reaching final tumor volumes more than twice as large as their ER+ LSCC counterparts regardless of estrogen supplementation. This was further borne out in a second LSCC xenograft model, which showed that natively ER+ LSCC silenced for the ESR1 gene behaved like ER− LSCC *in vivo*. shESR1-UM-SCC-12 xenografts grew to larger tumor sizes than either WT or E_2_-treated ER+ LSCC xenografts regardless of estrogen supplementation.

*In vitro* studies confirm this, with silencing ESR1 in ER+ LSCC reducing total p53 levels to those on par with E_2_-treated ER+ LSCC. This suggests that ERα loss mimics the effects of E_2_ treatment on ER+ LSCC, implying that the presence of ERα, independent of its role as an estrogen receptor, affects some anti-tumorigenic signaling, which is superseded in the presence of supra-physiological doses of E_2_. E_2_-ERα signaling has been well established as pro-tumorigenic^46^ in breast cancer, but the role of ERα independent of its actions as an E_2_ conduit has not been widely studied. However, other studies in breast and other hormonally response cancers have shown that loss of ER contributes to tumor aggression^51,52,58^.

Although ER expression profiles were not confirmed in the harvested xenograft, expression of ERα and ERβ isoforms pre-implantation suggests that both UM-SCC-12 and UM-SCC-11A have similar levels of ERα36 expression, but only UM-SCC-12 express ERα66. This is in line with observations from clinical and TCGA data, which suggest that increased ERα is associated with lower aggression in LSCC. It should also be noted that silencing ESR1 in UM-SCC-12 reduces both ERα66 and ERα36 expression in these cells, making it difficult to determine if the increase in aggression in shESR1-UM-SCC-12 is due to a reduction in ERα66, ERα36, or some combination of the two. The similar levels of ERα36 in UM-SCC-11A and UM-SCC-12 cells suggest that the increased aggression observed in shESR1-UM-SCC-12 cells was due to a reduction in ERα66 expression, but further studies are needed to determine which isoform(s) of ERα are involved in ERα-silencing induced increases in tumor aggression.

Survival data from head and neck cancer and LSCC patients further suggest that lack of ERα66 is associated with poorer prognoses, again in a manner similar to breast cancer. It is well known that loss of ERα66 correlates with reduced survival in breast cancer patients^59^, and similar trends were observed in our meta-analysis of recent head and neck cancer cohorts. Although all head and neck cancer patients show a slight correlation between increased ERα or ERβ expression and improved survival odds, this trend is not mirrored in laryngeal cancer, where ERα is the predominant determinate of ER-associated survival. It should be noted that the majority of the samples in this data set came from patients with stage 4 cancer (n = 81 of 140), suggesting that the prognostic value of ESR1 expression as a marker of LSCC aggression may exist independent of any association with late-stage cancer. It is also interesting to note the discrepancy between the whole head and neck cancer dataset and laryngeal cancer survival specifically. RNAseq data necessarily lacks information about post-transcriptional modification of transcribed proteins and can offer limited insights about splice variants and alternative isoforms of the traditional ERα and ERβ, many of which have been identified^19,60,61^. A lack of correlation between ESR1 and ESR2 expression may indicate a mechanistic difference in the ERα and ERβ membrane and cytosolic signaling pathways that could be dependent on alternative isoforms of both ERs.

The mechanism underlying the loss of ER expression in primary ER positive tumors has not yet been clarified. Genomic and posttranscriptional silencing mechanisms offer an explanation for the loss of ER expression. One proposed mechanism is the inactivation of ER gene transcription due to methylation of cytosine-rich areas termed CpG islands^62^. This mechanism has also been described in another hormone responsive tumor, endometrial cancer^63^. Similar silencing of ER gene transcription is the basis of the mechanism underlying the action of micro-RNA. The micro-RNA are a class of regulatory molecules that have been shown to control gene expression such as ERα in breast cancer^64^, and have been implicated in ovarian^65^, endometrial^66^ and laryngeal cancer^67^. An alternative genomic mechanism has been proposed, that entails the loss of heterogeneity in ER genes such as allelic loss in microsatellites located in regulatory regions of ER genes^68^, which was verified as an independent prognostic factor for relapse free survival^69^.

These mechanisms of loss of ER expression resemble the findings we have observed in laryngeal cancer. The indolent laryngeal cancer cells expressed both ERα66 and ERα36, and their ER profile resembled that of the epithelia adjacent the aggressive tumors, being part of a spectrum from precancerous lesions to low grade tumors. Stage 2 and 3 samples began to lose ERα66 and gain ERα36 expression, and the aggressive tumors were distinctly characterized by the loss of ERα66 and high expressing ERα36.

40% of ER positive and ER negative breast cancer cells express ERα36 in the plasma membrane^70^. High levels of this receptor’s expression correlate with an unfaborable prognosis. This correlation is unrelated to ER status and might serve as a novel marker for characterizing breast cancer in the clinic^71^. Membrane bound ERα36 and its action in mediating the response to E_2_ in ER negative tumors, as well as its potential to cause resistance to anti-estrogen therapeutics in ER positive tumors has led to increased clinical interest in this receptor. In our previous studies and in this recent study we have consistently identified high rates of ERα36 expression in laryngeal cancer cells^12,21^. Furthermore, in a previous study, we demonstrated that ERα36 activation resulted in increased angiogenic and metastatic factors and found a relationship between the amount of ERα36, VEGF, and lymph node metastasis in laryngeal cancer patients, indicating the metastatic role of ERα36^12^. In laryngeal cancer it seems that ERα36 has a pivotal role in tumorigenesis and tumor progression, with both indolent laryngeal cancer samples and epithelia adjacent to aggressive samples expressing high levels of ERα36.

The epithelia adjacent to all the tumors collected from Meir Hospital, Israel in our study expressed high levels of ERβ, and the level of expression of ERβ was maintained in the indolent tumor. In contrast the aggressive tumors from this sample set demonstrated a marked decline or full suppression of the expression of ERβ. These results indicate that ERβ confers protective effect in laryngeal cancer cells, an effect that has been reported previously in studies of head and neck carcinoma^13^. ERβ may inhibit the transcriptional response that ERα has to estrogens. The ratio of these two receptor types may impact cell sensitivity and response to E_2_^72^. It has been found that ERβ expression inhibited proliferation and cell invasion in breast cancer cells, thus offering some protective effect^73^. Further, ERβ expression has been clinically correlated with low grade tumors, low S phase fraction, negative axillary node status and most importantly increased likelihood to respond to hormonal therapy^74^. These findings coincide with our observation that advanced laryngeal tumors with metastasis to regional lymph nodes fully suppress the expression of ERβ. The relative conservation of ERβ was correlated with a more indolent tumor behavior. Up to 90% of triple negative breast cancers have been found to express ERβ^75^, the activation of which resulted in suppression of cell proliferation, presumably due to suppression of cyclin kinase 1 and 7, and blockage of cell cycle progression^76^. ERβ target genes have been postulated to regulate apoptosis and cell survival, development, growth, proliferation, and movement of the cell, as well as genes involved in cell cycle checkpoint and β-catenin pathways^75^. Knockdown of the ERβ expression in triple negative breast cancer cells increased the invasiveness of the cells about three fold^77^, while ERβ agonists had the opposite effect^78^, raising the possibility of their role in the treatment of these cancers.

A number of studies have identified geographic and racial disparities in ERα and ERβ expression in other hormonally responsive cancers, particularly breast cancer and prostate cancer. Geographic incidences of ER negative breast cancer are highly variable both within the U.S. and internationally^79–81^, and this disparity has been associated with higher incidences of ERα negative, ERβ positive, and triple-negative breast cancer in women of African descent^82–84^. The opposite trend has been observed in prostate cancer, with one study of 300 prostate cancer samples from African American (AA) and Caucasian American (CA) men identifying increased ERβ expression in tumor and precancerous samples from AA patients as compared to CA men^85^. However, both sets of patients had increased intra-tumor ERβ staining as compared to matched precancerous samples.

The proportion of ERα positive breast cancer incidence relative to the total diagnoses is similar for patients in the United States and Northern Israel^86,87^; however, our data suggest that ERβ positive cancer incidence may be more regionally localized. Other studies have reported high variability of ERβ expression in laryngeal cancer samples, similar to the trends we observed in our samples^88^. It is important to note that although western blots were conducted in a centralized lab, our samples were originally fixed and processed at two different institutions, which could have caused “batch processing effects” that could also account for the regional variation we observed in our samples.

It is possible that the wide variability of ERα and ERβ expression in our data could be indicative of variability in race and ethnicity among our sample sets, but additional demographic data on race and ethnicity were not available for our samples, limiting any conclusions from the analysis we performed. The two patient populations were chosen in order to validate the data. Unfortunately, when we separated the two groups, there was not enough power for statistical analysis. Our sample size was too small to draw conclusions concerning geographic variability of ERα and ERβ expression in laryngeal cancer, making further study needed to understand the potential of ERs as diagnostic and prognostic markers of laryngeal cancer.

The trend in ER levels in the histological specimens was not expected, but when the data were corrected to the receptor levels in the patient’s healthy normal tissue, the trend was very consistent. The results indicate that the level of the receptor was correlated with the stage of the disease.

## Conclusion

The findings of this study, together with our previous studies^12,19,21,40^, substantiate laryngeal cancer as a sex hormone responsive cancer that responds tumorigenically to the effects of E_2_. These E_2_ initiated-effects are mediated by the activation of different nuclear and membrane ERs, and their full impact on LSCC tumorigenicity is due to the interaction of different estrogen receptor pathways. The integrative, synergistic, or alternatively antagonistic effects of ERs cause variable response and reaction to E_2._ This is emphasized by the actions of the three central receptors, ERα66, ERα36 and ERβ, identified in laryngeal cancer cells. While E_2_ had a harmful effect increasing agression in cancers expressing ERα, it had no effect on either WT or silenced ER− LSCC *in vivo*. The splice variants of ERα have previously demonstrated synergistic deleterious effects in laryngeal cancer cell lines^21^, however the crosstalk between the different pathways is much more intricate, as the loss of the expression of ERα66 seems to confer the most aggressive phenotype, similar to that seen in breast cancer. Establishing the molecular and clinical characterization of the specific tumor and their precursors is crucial in order to optimize treatment and reduce adverse effects to the specific patient. Anti-estrogens, antagonists to ERα66 and ERα36, and agonists of ERβ might have a future role in the treatment of laryngeal cancers. Future work must be done to clarify the varying role of E_2_ in the pathogenesis and progression of laryngeal cancer, as well as to determine its potential role in the comprehensive treatment of these patients.

## Materials and Methods

### Cell culture

UM-SCC-12 cells and UM-SCC-11A cells were purchased from the Cancer Research Laboratory, Department of Otolaryngology/Head and Neck Surgery at the University of Michigan. Cells were maintained as previously described^21^. As noted previously, both cell lines expressed ERS1. However, UM-SCC-12 cells expressed transcriptionally active ERα66 and ERα36 whereas UMR-SCC-11A produced only ERα36^21^. Estrogen receptor profiles were also compared against established estrogen receptor profiles^89^. The two cell lines were further characterized as described below.

### Estradiol production

#### Aromatase activity

Confluent cultures of UM-SCC-11A and UM-SCC-12 cell monolayers were harvested and assessed for aromatase activity (Aromatase [CYP19A] Activity Assay Kit, Fluorometric, Biovision, Milpitas, CA) according to manufacturer’s instructions. Monolayers were also assessed for total protein content (Pierce 660nm Protein Assay, ThermoFisher, Waltham, MA), and aromatase activity was normalized to total protein.

#### 17β-estradiol parameter assay

UM-SCC-11A and UM-SCC-12 cells were cultured to confluence in hormone-free phenol-red-free media supplemented with 10% charcoal-dextran stripped fetal bovine serum (CD-FBS). At confluence, conditioned media were harvested and assessed for total 17β-estradiol content (Estradiol parameter assay kit, R&D Systems, Minneapolis, MN). Monolayers were washed twice in 1X PBS, lysed in 0.05% Triton X 100, and assessed for total double-stranded DNA content as described below. Total media 17β-estradiol was normalized to total double-stranded DNA content.

### Total p53 content

p53 is a tumor suppressor gene that can induce apoptosis. Total p53 levels were measured 24 hours post-treatment with a sandwich ELISA (Human Total p53 DuoSet® IC, R&D Systems, Minneapolis, MN). Cells were cultured to confluence in 24-well plates, then treated with fresh full media containing either 0 or 10^−7^ M E_2_ for 9 minutes. These media were removed by aspiration and fresh media were added to the cultures for 24 hours. Cultures were then washed twice in 1X PBS and harvested in 200μL of p53 lysis buffer according to manufacturer’s instructions, then assayed for total protein content (ThermoFisher, Pierce 660 nm Protein Assay), and normalized as previously described^21^.

### DNA quantification

Cultures were treated with 0 or 10^−7^ M E_2_ for 9 minutes followed by 24 hours in fresh media. Cell layers were washed with PBS and lysed with p53 lysis buffer. Total double-stranded DNA content was measured using the QuantiFluor® dsDNA System according to manufacturer’s instructions (Promega, Madison, WI).

### Production of UM-SCC-12 cells silenced for ESR1

Cells lines containing scramble control mRNA (TurboGFP Cat.# SHCOO4V, Millipore Sigma, Burlington, MA) and cells silenced for ESR1 (shESR1-UM-SCC-12) (Cat.# SHCLNV-NM_000125, Millipore Sigma) were created by transducing WT UM-SCC-12 cells with commercially available lentiviral shRNA plasmids and selecting the cells with 5μg/mL puromycin. Cells were maintained in full media containing 5μg/mL puromycin. Knockdown was assessed with western blots as described below.

### Tumor model

#### Subcutaneous xenograft laryngeal cancer mouse model

UM-SCC-11A (n = 16) and UM-SCC-12 (n = 16) cell lines were mixed with 1X DPBS (Thermo Fisher Scientific, Waltham, MA) and phenol-red free Cultrex basement membrane extract (BME, Trevigen, Gaithersburg, MD) to a concentration of 7.5 mg/mL BME and 10 million cells/mL. The resulting cell suspension was kept on ice and 100μL (1 million cells) was injected subcutaneously into the left flank of a 6-week old male NSG mouse (NOD.Cg-*Prkdc*^*scid*^ *Il2rg*^*tm1Wjl*^*/*SzJ, Cancer Mouse Models Core, Massey Cancer Center, Virginia Commonwealth University, Richmond, VA) to create a subcutaneous xenograft model of laryngeal cancer. At the time of cell injection, each animal was also subcutaneously implanted with a 0.96mg/60-day release E_2_ pellet or a corresponding placebo pellet to create four experimental groups: UM-SCC-11A + Placebo, UM-SCC-11A + E_2_, UM-SCC-12 + Placebo, UM-SCC-12 + E_2_ (n = 8 for each condition)^90^. Tumors were allowed to grow for 8 weeks, and tumor measurements were taken with digital calipers starting at week 2 until the end of the study. After 8 weeks, mice were euthanized by CO_2_ inhalation and cervical dislocation, and tumors were extracted and preserved on wet ice (<6 hours) until μCT analysis. After μCT analysis, tumors were fixed in formalin and histologically analyzed as described below. All animal experiments were conducted in full compliance with the recommendations for the Care and Use of Laboratory Animals of the National Institutes of Health under a protocol approved by the Virginia Commonwealth University Institutional Animal Care and Use Committee (protocol number AD10000675).

In a second experiment, WT UM-SCC-12 and shESR1-UM-SCC-12 subcutaneous xenografts were created as described above (n = 16 for each cell line). Animals were subcutaneously implanted with a 0.96mg/60-day release E_2_ pellet or a corresponding placebo pellet as described above to create four experimental conditions: WT UM-SCC-12 + Placebo, WT UM-SCC-12 + E_2_, shESR1-UM-SCC-12 + Placebo, shESR1-UM-SCC-12 + E_2_ (n = 8 for each condition). *In vitro* studies showed that scrambled control UM-SCC-12 cells responded to E_2_ similar to WT cells. Therefore to preserve animal life, xenografts with scrambled control UM-SCC-12 cells were not created for this experiment. Tumors were monitored for 8 weeks until harvest. Animals were euthanized as described above and tumors were fixed in formalin.

#### μCT analysis

After harvest, tumors were preserved for less than 6 hours on wet ice and scanned with a Bruker Skyscan 1173 μCT at 55kV and 70μA at a resolution of 560 × 560 pixels with an image pixel size of 40.26 μm, an exposure time of 125ms and a rotation step of 0.8 degrees^91^. NRecon software version 1.6.10.4 (Kontich, Belgium) with a smoothing kernel of 0 and a beam hardening correction of 20% was used to reconstruct and analyze the tumors with a standard reconstruction protocol. Total tissue volume approximated total tumor volume.

#### Histology

After harvest, xenograft tumors were fixed in 10% neutral formalin for 7 days, processed, and embedded in paraffin. Samples were then sectioned into 4μm thickness and stained with haemotoxylin and eosin (H&E) as previously described^92^. Slides were imaged at 10X and 40X (Zeiss AxioVision Microscope, Carl Zeiss, Oberkuchen, Germany).

### Clinical sample acquisition

Frozen tissue samples and prepared H&E slides were acquired from Meir Hospital, Kfar Saba, Israel under Institutional Review Board (IRB) protocol #0497-13-RMC or from Massey Cancer Center, Virginia Commonwealth University (VCU), Richmond, Virginia, USA under an anonymization agreement in compliance with the Office for Human Research Protections’ “Guidance on Research Involving Coded Private Information or Biological Specimens”. Information on patient gender, age, tobacco and alcohol use, and tumor location (glottic/supraglottic), was provided by respective institutions (Tables 1 and 3). A value of ‘0’ indicates no or infrequent use of tobacco or alcohol products as reported by the patient; a value of ‘1’ indicates self-reported regular, heavy use of tobacco or alcohol products (Table 1).

**Table 3:** Summary of clinical laryngeal squamous cell carcinoma samples.

Number of Patients	14
Mean Age	65.6 ± 7.59
Female	1
Male	13
Supraglottic	6
Glottic	8
Heavy Tobacco User	13
Heavy Alcohol Consumer	5

### Estrogen receptor analysis via western blots

#### Preparation of lysates

For samples obtained from Meir Hospital, Israel, tissue pieces were weighed, and >10mg of tissue was minced into pieces <1 mm^3^ with a No.11 scalpel blade. Tissue pieces were homogenized with a Dounce tissue grinder in Nonidet p-40 (NP-40) containing 5mM NaF and 20μL of protease inhibitor cocktail (Sigma-Aldrich #P3480) per mL of NP-40. After homogenization, samples were sonicated on ice at 40 amperes for 5 seconds to obtain tissue lysates as previously described^93,94^.

For samples from the VCU Tissue and Data Acquisition and Analysis Core Laboratory (TDAAC), frozen tissue shavings were obtained and washed twice with 2 mL of 1X PBS containing 40μL of protease cocktail inhibitor. Samples were vortexed and centrifuged at 1000 rpm for 5 minutes between each wash. Tissue pellets were then resuspended in 200μL of RIPA buffer and sonicated on ice at 40 amperes for 5 seconds to obtain tissue lysates.

To assess ERα66 and ERα36 knockdown in shESR1-UM-SCC-12, WT UM-SCC-12, and Scramble Control UM-SCC-12 cells, cultures were grown in T-75 flasks (n = 1 flask per cell line) until confluent, washed twice with 1X phosphate buffered saline (PBS), and cell layers were lysed with 300μL RIPA as previously described^95^.

Tissue and cell lysates were incubated on ice for 30 minutes and centrifuged at 13000g for 20 minutes at 4C. Supernatants were saved and assayed for total protein content and used in western blots as described below.

#### Western blots

50 μL of tissue or cell lysate containing 8–35μg protein were loaded onto 4–20% Mini-PROTEAN^®^TGX™ precast polyacrylamide gels (Bio-Rad, Hercules, CA). Proteins were transferred to low-fluorescence PVDF membranes using a Trans-Blot^®^ Turbo™Transfer System (Bio-Rad). Membranes were blocked for 1 hour at room temperature in odyssey blocking buffer (LI-COR) and then incubated with antibodies against GAPDH (mouse monoclonal, Millipore, Burlington, MA) and ERα (rabbit polyclonal, Chi Scientific, Maynard, MA), which detects ERα66 and ERα36, or ERβ (rabbit polyclonal, Abcam, Cambridge, United Kingdom) for 24 hours at 4 °C. Membranes were then incubated with IRDye 700CW (goat anti mouse) and IRDye 800CW (goat anti-rabbit) conjugated secondary antibodies (LI-COR) for 45 minutes at room temperature and imaged using the LI-COR Odyssey® CLx Infrared Imaging System. Western blots were carried out at a central institution (VCU) to minimize variation and repeated once (a total of two experiments per assay) with 1 replicate per sample in each experiment. Signal intensity for proteins of interest (ERα66, ERα36, and ERβ) was normalized to GAPDH. Where no signal was detected, samples were considered to have ‘0’ signal intensity and marked with ‘N.D.’, or no signal detected, on graphs (Figs. S1 and S2).

Normalized signal intensity for ERα66, ERα36, and ERβ in each tumor sample was grouped by stage (stage 1, stage 2 & 3, stage 4) and graphed (Fig. 4A–C); or normalized to corresponding signal intensities in adjacent epithelial tissue (Fig. 4D). A normalized ratio of greater than 1 was recorded with a blue upward-pointing arrow **↑**, and a ratio less than 1 was recorded with a red downward-pointing arrow ↓. To generate heat maps, log_10_ of normalized signal intensities of tumor or normal epithelial tissue was calculated and graphed using the Graphpad Prism 7 Heatmap generator. Samples were grouped by stage and arranged in order of increasing cancer aggression (top-bottom).

### Accessing ESR1 and ESR2 gene transcript expression data

ESR1 and ESR2 expression data were publically available and were obtained from The Cancer Genome Atlas (TCGA) cohort (Project Id: TCGA-HNSC) (n = 525 patients) (https://portal.gdc.cancer.gov/projects/TCGA-HNSC) using University of California Santa Cruz (UCSC) Xena (http://xena.ucsc.edu/)^96,97^. In brief, ESR1 and ESR2 expression data were downloaded from Xena and only samples taken from primary solid head and neck squamous cell carcinomas were included in overall head and neck cancer analyses. Samples were further separated by neoplasm tissue type, and only primary LSCC samples were included in the second analysis. Overall survival (OS) was defined as time of death by any cause and capped at fifteen years. Kaplan-Meir curves of OS were generated by GraphPad Prism v5.0 (GraphPad Software Inc.). Patients were grouped according to median gene expression as described previously^98^.

### Statistical analysis

μCT tumor volumes were compared with one-way ANOVA analysis with Tukey’s correction. Tumor volume measurements over time were analyzed with a repeated-measures ANOVA. *Ex vivo* western blots were grouped by stage and analyzed with one-way ANOVA analysis with Tukey’s correction to compare between stages and two-way ANOVA analysis with Bonferroni post-tests between stages to determine significance between precancerous and tumor tissue. Tumor protein expression for ERα66, ERα36, and ERβ was normalized to matched precancerous tissue and graphed as treatment/control (D). Two-way ANOVA analysis with Bonferroni post-tests between stages was done to determine significance between stages. Statistical analysis was performed by using GraphPad Prism v5.0. Comparison of high and low ESR survival curves was done with a Log-rank Mantel-Cox χ^2^ test. For all studies, p-values < 0.05 were considered significant.

## Supplementary information

Supplemental Figures and Tables.

Dataset for Figures 1-6.

## Data Availability

All data generated or analyzed during this study are included in this published article (and its Supplementary Information files).
