# Supplementary material for: Loss of Estrogen Receptors is Associated with Increased Tumor Aggression in Laryngeal Squamous Cell Carcinoma
Source: Sci Rep. 2020 Mar 6;10:4227. doi: 10.1038/s41598-020-60675-2 (PMC7060328; doi:10.1038/s41598-020-60675-2)
Supplement: Supplementary file 1 — Supplemental Figures and Tables. [file 41598_2020_60675_MOESM1_ESM.pdf]

Title: Loss of Estrogen Receptors is Associated with Increased Tumor Aggression in Laryngeal Squamous Cell Carcinoma

Authors: Anjali Verma, B.S.[1*], Nofrat Schwartz, M.D.[2,3,4*], David J. Cohen, M.D.[1], Vaidehi Patel, B.S.[1,5], Benny Nageris, M.D.[2,3], Gideon Bachar, M.D.[3,6], Barbara. D. Boyan, Ph.D.[1,7]*, and Zvi Schwartz, D.M.D., Ph.D.[1,8]

Affiliations: [1]Department of Biomedical Engineering, Virginia Commonwealth University, Richmond, VA; [2]Department of Otolaryngology, Meir Hospital, Kfar Saba, Israel; [3]Sackler Faculty of Medicine, Tel Aviv University, Tel Aviv, Israel; [4]Department of Otolaryngology – Head and Neck Surgery and Neurosurgery, University of North Carolina School of Medicine, Chapel Hill, NC; [5]University of Pittsburgh School of Medicine, Pittsburgh, PA; [6]Department of Otolaryngology Head and Neck Surgery, Rabin Medical Center, Petah-Tikvah, Israel; [7]Department of Biomedical Engineering, Georgia Institute of Technology, Atlanta, GA; [8]Department of Periodontics, University of Texas Health Science Center at San Antonio, San Antonio, TX

*These authors contributed equally to the publication of this manuscript

Supplemental Figures

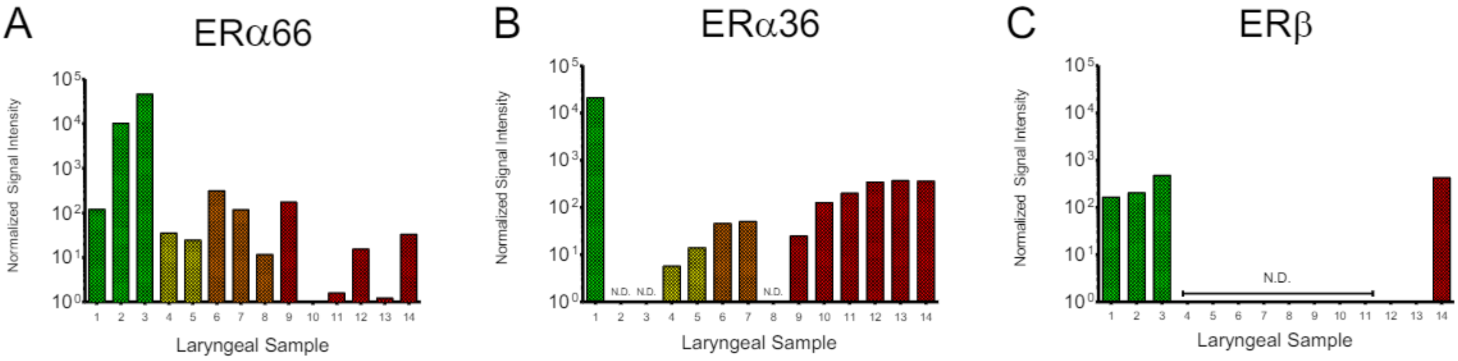

Supplemental Figure 1: Quantification of western blots for ERα66 (A), ERα36 (B), and ERβ (C) for individual clinical tumor samples. Y-axes are given as log scales. Samples are color-coded according to clinical stage. Stage 1 samples are green, stage 2 yellow, stage 3 orange, and stage 4 red. N.D. = no signal detected.

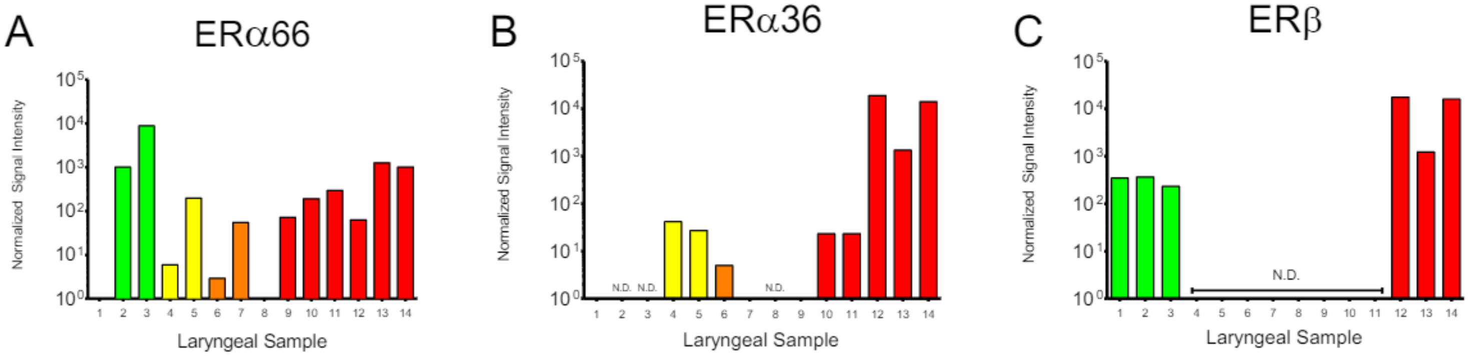

Supplemental Figure 2: Quantification of western blots for ERα66 (A), ERα36 (B), and ERβ (C) for normal epithelia adjacent to clinical tumor samples. Y-axes are given as log scales. Samples are color-coded according to clinical stage. Stage 1 samples are green, stage 2 yellow, stage 3 orange, and stage 4 red. N.D. = no signal detected.

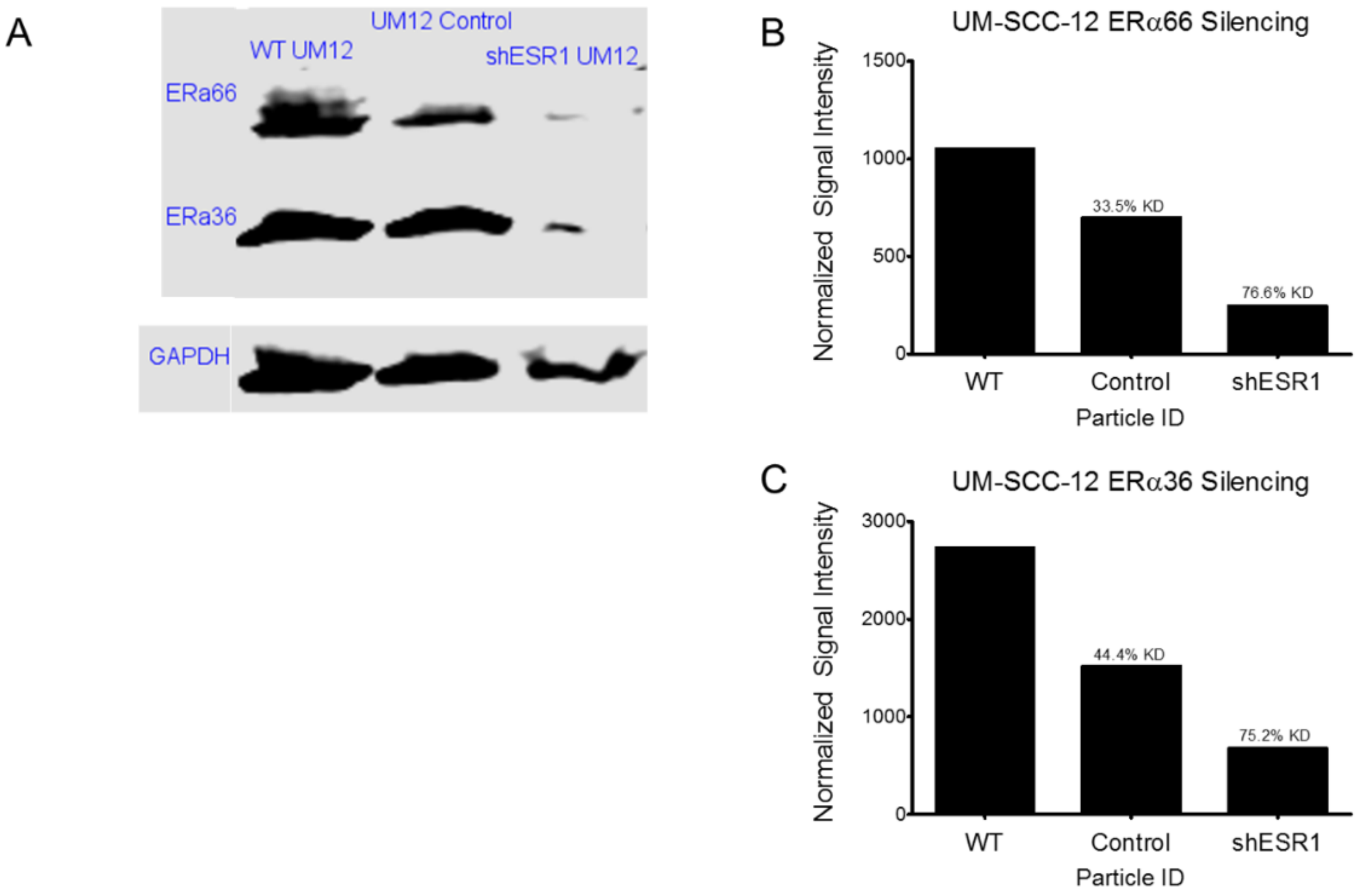

Supplemental Figure 3: A representative western blot (A) showing ERα expression in WT, scramble-control, and ESR1-silenced UM-SCC-12 cell lines. GAPDH was used as a protein loading control. ERα66 (B) and ERα36 (C) protein expression were normalized to GAPDH and quantified by normalized signal intensity. N=1.

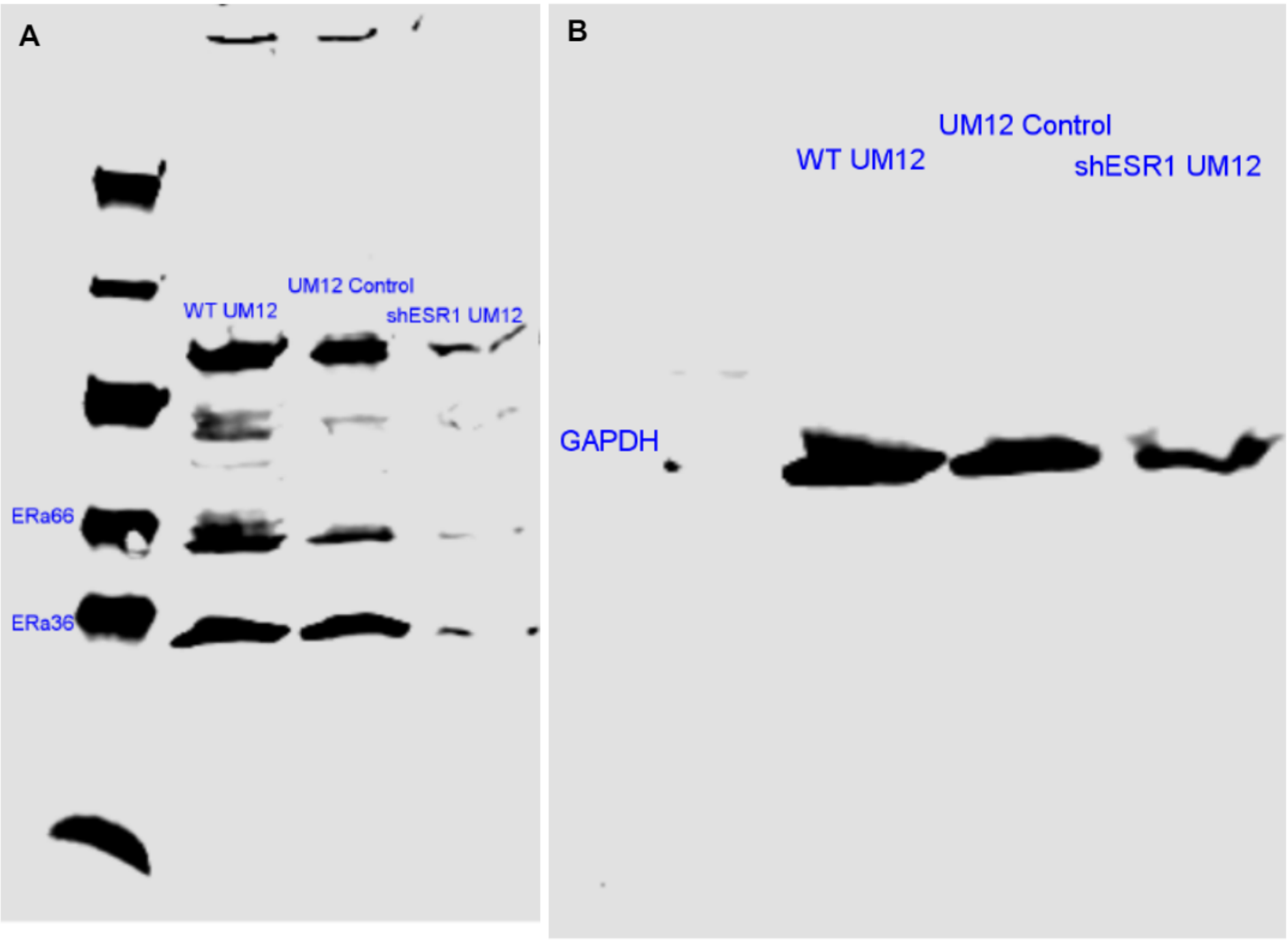

Supplemental Figure 4: Whole western blot of WT, scramble-control, and ESR1-silenced UM-SCC-12 cells probed with (A) an all-ER antibody and (B) a GAPDH antibody.

Supplemental Tables

Table S1 Xenograft models of LSCC tumor growth curve: linear fit, weeks 2-8

	11A Control	11A 17b-Estradiol	12 Control	12 17b-Estradiol
Best-fit values				
Slope	346.1 ± 46.88	428.2 ± 47.74	63.91 ± 12.24	164.9 ± 19.50
Y-intercept when X=0.0	-1091 ± 280.8	-1254 ± 263.7	47.39 ± 65.68	-352.4 ± 106.0
X-intercept when Y=0.0	3.152	2.929	-0.7415	2.138
1/slope	0.002890	0.002335	0.01565	0.006065
95% Confidence Intervals				
Slope	249.3 to 442.8	331.5 to 525.0	39.31 to 88.51	125.9 to 203.9
Y-intercept when X=0.0	-1671 to -511.3	-1789 to -719.8	-84.66 to 179.4	-564.6 to -140.3
X-intercept when Y=0.0	2.001 to 3.866	2.103 to 3.519	-4.449 to 0.9814	1.085 to 2.844
Goodness of Fit				
R square	0.6943	0.6850	0.3530	0.5478
Sy.x	417.0	612.1	180.6	306.4
Is slope significantly non-zero?				
F	54.50	80.46	27.28	71.48
DFn, DFd	1.000, 24.00	1.000, 37.00	1.000, 50.00	1.000, 59.00
P value	< 0.0001	< 0.0001	< 0.0001	< 0.0001
Deviation from zero?	Significant	Significant	Significant	Significant
Data				
Number of X values	7	7	7	7
Maximum number of Y replicates	5	7	8	9
Total number of values	26	39	52	61
Number of missing values	44	31	18	9

Table S2 Xenograft models of LSCC tumor growth curve: exponential fit, weeks 2-8

Exponential growth equation	11A Control	11A 17b-Estradiol	12 Control	12 17b-Estradiol
Best-fit values				
Y0	44.95	15.50	163.4	54.62
k	0.4723	0.6563	0.1533	0.3782
Tau	2.117	1.524	6.524	2.644
Doubling Time	1.468	1.056	4.522	1.833
Std. Error				
Y0	24.49	7.819	38.50	21.42
k	0.07290	0.06586	0.03665	0.05400
95% Confidence Intervals				
Y0	-5.601 to 95.49	-0.3556 to 31.35	86.01 to 240.8	11.77 to 97.48
k	0.3218 to 0.6227	0.5228 to 0.7898	0.07960 to 0.2270	0.2701 to 0.4862
Tau	1.606 to 3.108	1.266 to 1.913	4.406 to 12.56	2.057 to 3.702
Doubling Time	1.113 to 2.154	0.8776 to 1.326	3.054 to 8.708	1.426 to 2.566
Goodness of Fit				
Degrees of Freedom	24	37	50	59
R square	0.7679	0.8778	0.3106	0.5898
Absolute Sum of Squares	3.168e+006	5.376e+006	1.738e+006	5.025e+006
Sy.x	363.3	381.2	186.5	291.8
Number of points				
Analyzed	26	39	52	61

Table S3 Xenograft models of LSCC tumor growth curve: linear fit, weeks 4-8

	11A Control	11A 17b-Estradiol	12 Control	12 17b-Estradiol
Best-fit values				
Slope	441.2 ± 69.19	684.9 ± 79.54	19.09 ± 22.63	209.0 ± 37.80
Y-intercept when X=0.0	-1728 ± 443.0	-2958 ± 503.3	343.3 ± 140.8	-638.4 ± 234.5
X-intercept when Y=0.0	3.917	4.318	-17.99	3.055
1/slope	0.002267	0.001460	0.05239	0.004785
95% Confidence Intervals				
Slope	296.9 to 585.5	521.4 to 848.4	-26.93 to 65.11	132.7 to 285.3
Y-intercept when X=0.0	-2653 to -804.3	-3992 to -1923	56.95 to 629.7	-1112 to -164.9
X-intercept when Y=0.0	2.664 to 4.606	3.627 to 4.784	-infinity to -0.8896	1.221 to 3.969
Goodness of Fit				
R square	0.6703	0.7404	0.02050	0.4213
Sy.x	417.2	574.1	194.5	350.3
Is slope significantly non-zero?				
F	40.66	74.15	0.7115	30.57
DFn, DFd	1.000, 20.00	1.000, 26.00	1.000, 34.00	1.000, 42.00
P value	< 0.0001	< 0.0001	0.4048	< 0.0001
Deviation from zero?	Significant	Significant	Not Significant	Significant
Data				
Number of X values	5	5	5	5
Maximum number of Y replicates	5	7	8	9
Total number of values	22	28	36	44
Number of missing values	28	22	14	6
